# Prevalence of Iron Deficiency Anemia in Gynecological Cancer Patients Undergoing Treatment: A Two-Institution Retrospective Study

**DOI:** 10.7759/cureus.74163

**Published:** 2024-11-21

**Authors:** Taro Yagi, Yasuto Kinose, Mai Koizumi, Tadashi Iwamiya, Futa Ito, Satoshi Kubota, Kenjiro Sawada, Tadashi Kimura, Masahiko Takemura, Ken-ichirou Morishige

**Affiliations:** 1 Department of Obstetrics and Gynecology, Osaka General Medical Center, Osaka, JPN; 2 Department of Obstetrics and Gynecology, Graduate School of Medicine Faculty of Medicine, Osaka University, Suita, JPN

**Keywords:** absolute iron deficiency, chemotherapy-induced anemia, functional iron deficiency, gynecologic cancer, iron deficiency anemia, iron replacement therapy

## Abstract

Background: Anemia in patients with cancer negatively affects their quality of life and cancer outcomes. However, most patients with chemotherapy-induced anemia (CIA) are not appropriately evaluated or treated, and the prevalence of iron deficiency anemia (IDA) in CIA remains unclear.

Methods: We retrospectively reviewed the electronic records of patients with gynecological cancer in two tertiary hospitals, between March 2023 and July 2023, and evaluated their anemia status.

Results: We identified 54 patients with CIA, and IDA was found in 74% (40/54) of patients with CIA, including 4% (2/54) with absolute iron deficiency (transferrin saturation (TSAT) < 20% and ferritin < 30 ng/mL), 63% (34/54) with functional iron deficiency (TSAT < 50% and 30 ng/mL < ferritin < 500 ng/mL), and 7% (4/54) with possible functional iron deficiency (TSAT < 50% and 500 ng/mL < ferritin < 800 ng/mL).

Conclusions: We found that 74% of patients with CIA under gynecologic cancer treatments were IDA in this study.

## Introduction

Chemotherapy-induced anemia (CIA) is often observed during cancer treatment, including chemotherapy with or without radiation therapy. According to an extensive survey study, the European Cancer Anemia Survey (ECAS), CIA with less than 10.0 g/dL of hemoglobin (Hb) was found among 39% of 15,367 patients undergoing chemotherapy [1]. Anemia in patients with cancer is associated with a wide range of symptoms, including fatigue, dizziness, palpitations, dyspnea, anorexia, and heart failure, resulting in poor quality of life (QOL). However, such symptoms depend on the degree of anemia, onset rapidity, and existing comorbidities. Although controversial, many studies have reported that anemia is an independent unfavorable factor that adversely affects the survival of patients with cancer, especially among those treated with chemotherapy and radiotherapy, which require adequate oxygen levels [1]. In gynecologic oncology, cervical cancer is generally considered radiosensitive, and low Hb levels in patients with cervical cancer caused by anemia due to chronic disease, vaginal bleeding, or concurrent chemotherapy have been associated with poor local control rates after radiotherapy [2]. Several studies have reported that red blood cell transfusion before radiation in patients with cervical cancer and anemia is associated with a higher local control rate and overall survival [2]. Therefore, it is essential to address CIA during cancer therapy.

The causes of CIA are multifactorial, including blood loss, hemolysis, myelosuppression, chronic inflammation, and nutritional deficiency, and are often difficult to identify in detail [1]. The National Comprehensive Cancer Network (NCCN) guidelines classify CIA into four groups (absolute iron deficiency, functional iron deficiency, possible functional iron deficiency, and no iron deficiency) based on transferrin saturation (TSAT) and ferritin values [3]. The NCCN recommends that intravenous or oral iron replacement therapy (IRT) should be considered for absolute iron deficiency, whereas IRT with erythropoiesis-stimulating agents (ESAs) should be considered for functional iron deficiency. IRT is also an optional treatment for patients with functional iron deficiencies. In line with the NCCN guidelines, the European Society of Medical Oncology (ESMO) Clinical Practice Guidelines [4] showcase the management of CIA in patients with solid or hematological malignancies. According to the ESMO guidelines, those with Hb 10-11 g/dL are recommended to be administered 1,000 mg of iron intravenously if TSAT is less than 20% or serum ferritin is less than 100 ng/mL, whereas those with Hb 8-10 g/dL without vitamin B12 and folate deficiency are recommended to receive intravenous iron if serum ferritin is less than 100 ng/mL or TSAT is less than 20% [4]. However, during ECAS surveillance, only 39% of patients with CIA are treated with ESAs or blood transfusions [1]. Hufnagel et al. reported that anemia was observed in 21.7% of gynecologic cancers with a median Hb of 9.8 g/dL; however, only 36% of patients with anemia underwent evaluation for iron deficiency anemia (IDA) or anemia of chronic disease [5]. Blood transfusion was the most common intervention (79%), followed by IRT, concluding that compliance with the NCCN guidelines for the evaluation and treatment of CIA is low [5]. Collectively, these data suggest that there is room for improvement in the evaluation and management of CIA in patients with gynecological cancer.

Except for several articles [6-8], there is insufficient epidemiological data to evaluate whether anemia is associated with iron deficiency in patients with cancer, especially in the gynecologic field. Here, we aimed to retrospectively evaluate CIA in patients with gynecological cancer at two institutions.

## Materials and methods

This retrospective study was conducted at two institutions, Osaka General Medical Center and Osaka University Hospital, Japan. This study was approved by the ethics committees of the institutions (registration number: 2022-101, Osaka General Medical Center; approval number: 23267, Osaka University Hospital). Informed consent was obtained from all patients using an opt-out approach posted on the hospital websites. We reviewed the electronic records of patients with gynecological cancer who visited the outpatient department or were hospitalized in one of the two institutions between March 2023 and July 2023. The gynecological cancers in this study included ovarian, fallopian tube, peritoneal, cervical, endometrial, and other cancers (including leiomyosarcoma of the uterus and cancer of unknown primary origin showing gynecologic cancer markers on histology). Extracted data included age, disease, treatment regimen, and blood sample test results, including Hb, ferritin, and TSAT values. Patients who received ongoing medical treatment for gynecological cancer below 12.0 g/dL of Hb value were included. Moreover, we classified patients with CIA according to the NCCN guidelines [3]: absolute iron deficiency, TSAT < 20% and ferritin < 30 ng/mL; functional iron deficiency, TSAT < 50% and 30 ng/mL < ferritin < 500 ng/mL; possible functional iron deficiency, TSAT < 50% and 500 ng/mL < ferritin < 800 ng/mL; and no iron deficiency, TSAT ≥ 50% or ferritin > 800 ng/mL.

## Results

We identified 54 patients with CIA and gynecological cancer during medical treatment (n = 21 at Osaka General Medical Center and n = 33 at Osaka University Hospital) (Table 1). The mean age of the participants was 64.7 ± 9.7 years. The number of patients with ovarian/fallopian tube/peritoneal, cervical, uterine, and other cancers was 39, nine, four, and two, respectively. Thirty-nine percent (21/54) of the participants received a platinum-containing regimen (carboplatin plus paclitaxel, bevacizumab (BEV), and pembrolizumab (Pem); carboplatin plus paclitaxel and BEV; carboplatin plus paclitaxel and Pem; carboplatin plus paclitaxel and epirubicin; carboplatin plus paclitaxel; carboplatin plus docetaxel (DTX) and BEV; carboplatin plus DTX; and cisplatin plus nogitecan hydrochloride (topotecan)), which was administered as first-line standard-of-care chemotherapy for adjuvant setting after surgery (six patients) or advanced/recurrent stages (15 patients). Among those who were treated with a platinum-free regimen (pegylated liposomal doxorubicin (PLD) plus BEV, DTX plus BEV, gemcitabine (GEM) plus DTX, PLD, irinotecan, GEM, and doxorubicin), 17% (9/54) had metastatic or recurrent disease. The median treatment duration of all the participants was 94 days.

**Table 1:** Participant characteristics and results Poly (ADP-ribose): polymeric adenosine diphosphate ribose; IQR: interquartile range

	N = 54
Characteristics	
Age (year) (mean ± standard deviation)	64.7 ± 9.7
Disease, N (%)	
Ovarian cancer, fallopian tube cancer, peritoneal cancer	39 (72)
Cervical cancer	9 (17)
Uterine cancer	4 (7)
Others	2 (4)
Stage, N (%)	
I	8 (15)
II	3 (5)
III	21 (39)
IV	22 (41)
Treatment, N (%)	
Platinum-containing regimen	21 (39)
Nonplatinum agent	9 (17)
Olaparib plus bevacizumab	10 (18)
Poly (ADP-ribose) polymerase inhibitor single agent (olaparib or niraparib)	10 (18)
Immuno-checkpoint inhibitor (pembrolizumab)	3 (6)
Bevacizumab single-agent	1 (2)
Median treatment duration (days) (IQR)	94 (40, 184)
Platinum-containing regimen	69 (26, 118)
Nonplatinum agent	52 (19, 75)
Olaparib plus bevacizumab	162 (95, 262)
Poly (ADP-ribose) polymerase inhibitor single agent (olaparib or niraparib)	162 (108, 522)
Immuno-checkpoint inhibitor (pembrolizumab)	51 (41, 238)
Bevacizumab single-agent	44 (44, 44)
Iron replacement therapy	8 (15)
Erythropoiesis-stimulating agent	0 (0)
Results	
Median hemoglobin (g/dL) (IQR)	10.7 (9.7, 11.4)
Median ferritin (ng/mL) (IQR)	178 (69, 433)
Median transferrin saturation (%) (IQR)	27.5 (18.8, 40.8)
Classification of anemia, N (%)	
Absolute iron deficiency	2 (4)
Functional iron deficiency	34 (63)
Possible functional iron deficiency	4 (7)
No iron deficiency	14 (26)

**Figure 1:**
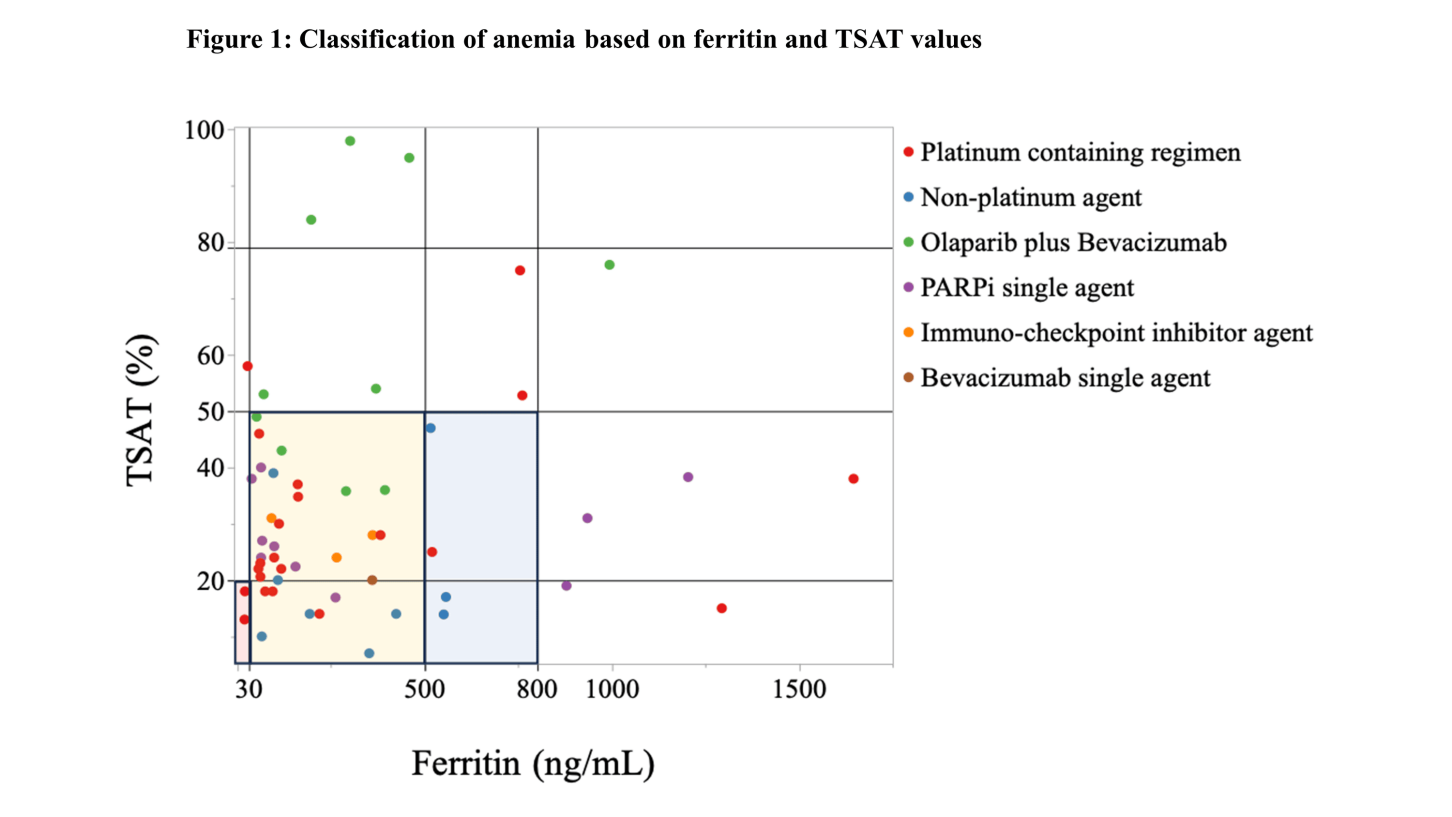
Classification of anemia based on ferritin and transferrin saturation (TSAT) values. Scattered plotting was performed based on ferritin and TSAT values. Each plot is colored according to the treatment. Based on the National Comprehensive Cancer Network (NCCN) guideline classification, the red-, yellow-, and blue-shaded areas represent absolute iron deficiency anemia, functional iron deficiency anemia, and possible functional iron deficiency anemia, respectively PARPi: poly (ADP-ribose) polymerase inhibitor

Regarding hematologic results, the median Hb was 10.7 g/dL (interquartile range (IQR) 9.7, 11.4) (Table 1). The median ferritin level was 178 ng/mL (IQR 69-433), and the median TSAT was 27.5% (IQR 18.8, 40.8). Regarding the classification of anemia, there were two patients (4%) with absolute, 34 patients (63%) with functional, and four patients (7%) with possible functional IDA (Table 1 and Figure 1). Among the 21 patients who were under treatment with a platinum-containing regimen, two patients (10%), 13 patients (62%), and one patient (5%) had absolute, functional, and possible functional IDA, respectively. However, in the 20 patients who had been administered a polymeric adenosine diphosphate ribose (poly (ADP-ribose)) polymerase inhibitor (PARPi) (olaparib plus BEV and PARPi single agent), absolute, functional, and possible functional IDA involved 0 (0%), 11 (55%), and 0 (0%) patients, respectively.

## Discussion

IDA was found in 74% of gynecological cancer patients with CIA who were undergoing treatment (Table 1 and Figure 1). We observed a relatively higher prevalence: according to a report that represents approximately 30% of patients with gynecological cancer, 63.2% of patients with pancreatic cancer, 52.2% of patients with colorectal cancer, and 51.3% of patients with lung cancer had IDA [8]. As the criteria for IDA in the report [8] were only based on TSAT < 20% and the population that contained functional IDA and possible functional IDA was not thoroughly evaluated, the frequency of IDA might be underestimated.

ESAs and blood transfusion therapies are commonly used to treat CIAs. However, IRT, vitamin B12 supplementation therapy, and folate supplementation therapy are less popular, partly because most patients with CIA have not been adequately evaluated [1,5]. Moreover, in Japan, ESAs are not covered by insurance because of concerns about their possible negative impact on tumors, and the only active treatment for CIA is often thought to be blood transfusion therapy [9]. If blood transfusion is not indicated or desired by patients, the dose of chemotherapy may be reduced, or its administration may be delayed. This may eventually lead to a decreased relative dose intensity (RDI), resulting in reduced efficacy. In particular, those who were treated with PARPi (olaparib or niraparib, which were approved in 2018 and 2020, respectively, by the universal health insurance system in Japan) are known to have a high frequency of anemia as an adverse event (39% in olaparib and 48% in niraparib treatment from real-world data) [10] and a high risk of attenuated RDI. RDI is associated with tumor prognosis [11], whereas CIA affects patient QOL [1]. Thus, oncologists should pay more attention to the treatment of the CIA.

Recently, IRT has become an effective treatment for CIA [12]. The NCCN recommends a total dose of 1,000-1,500 mg of parenteral iron products to treat absolute or functional IDA in patients with cancer receiving ESAs [3]. Moreover, ESMO offers intravenous iron (1,000 mg) to patients with cancer or IDA [4]. Furthermore, IRT alone has been suggested to be effective for IDA in patients with CIA. It could reduce the rate of blood transfusion in the field of gynecologic oncology [13,14].

Given that our study revealed that IDA accounted for 74% of CIA in all patients and 55% of CIA in those who received PARPi-containing treatment, IRT alone could be considered a promising therapy for CIA. However, few studies have evaluated the cause of CIA based on ferritin and TSAT levels, according to the NCCN [3] or ESMO [4] guidelines. Thus, the population with CIA that genuinely benefits from IRT and its efficacy remains unclear. In the future, we plan to examine whether IRT monotherapy is effective for absolute IDA and functional IDA based on the criteria of the NCCN guidelines for patients with gynecological cancer in a prospective observational study.

This study has several limitations. Owing to the small number of participants in the present study and the heterogeneity of the patients' backgrounds, including the cancer type, stage, and treatment regimen, the results might be biased. In addition, we did not record the total number of patients that were screened for anemia. Therefore, more extensive studies are required to confirm the results of this study.

## Conclusions

Among the patients with anemia and gynecological cancer who were undergoing chemotherapy, we found that 74% of the patients were classified as having absolute, functional, or possibly functional IDA. There is room for improvement in the evaluation and management of CIA in patients with gynecological cancer. Further treatment studies are needed to evaluate whether IRT improves QOL in patients undergoing cancer treatment and outcomes for patients with cancer in gynecologic oncology.
